# Relations Existing between the Clerical and Medical Prefessions

**Published:** 1848

**Authors:** 


### Part 3.—Selections.

### ARTICLE I.

*Relations existing between the Clerical and Medical Professions—the Christian Examiner and the Hydropathic delusion.* Communicated for the Boston Medical and Surgical Journal.

A recent number of this Journal gives an account of a vote introduced, and laid on the table for future consideration, at the meeting of the Connecticut Medical Society, the intent of which was to rescind that part of the code of medical ethics or usage by which physicians have declined compensation from ministers and their families for professional services. The innovation was based and defended on the ground that the deportment and feelings of the clergy, as a body, had broken off and annihilated that mutual relation of respect and support, on which so kindly a consideration had been so long sustained in New England. This regard to the ministers of the gospel is still more honorable to the physicians, when it is recollected that the service was from the poorer and worse compensated servant of the public, towards the less laborious and better paid; that is, if comparisons can be predicated between classes, both of which are so meagerly provided for as the country brethren.

A writer in a still earlier number, that of February 23d ult., gave some strange—probably before unsuspected—facts and statistics, demonstrating that colleges, always under clerical control and influence, had, from their very origin, treated the medical calling with a studied neglect, contempt, and omission—so palpable, so universal, as not to be accounted for or explained, on the idea of accident or mere coincidence. The physician has borne it all with "that sufferance which is the badge of all our tribe." This writer demonstrated, by a reference to triennial catalogues and other documentary publications of the colleges themselves, that medical men never had either lot or part in the boards of trustees, examining bodies, or as recipients of the higher honors, within their trust. In fact, in eighty members of trustee boards, two only are physicians. And in several of the colleges, no medical trustee had ever been appointed. Physicians, elevated to the chair of state, *ex officio*, alone formed the exception of medical men in this connection.

It would seem, from these indications, that the attention, the self respect, and the *esprit du corps* of our body have begun to be aroused. The time is approaching when medical men will examine and decide the question whether they are to be treated by the members of a cognate liberal profession as their equals in virtue, intelligence, and aim; whether there shall be that mutual support of callings, neither of which surely, in these latter days of reforms and innovations, can be in imminent danger of being injured by too profuse demonstrations of general respect and consideration.

Which party, in the present antagonistic position, is responsible for the earliest aggression it is not needful to decide, nor which party can least afford this internecine war against each other's standing and interest. Our medical brethren in the interior, who have long borne in silence the officious interference of the clergy in determing a professional preference "amongst their people," in injudicious ministrations, in the sick chamber, irrespective of the judgment or wishes of the physician attending, and in partisan efforts towards introducing "a member of our society," into circles of practice already overcrowded, can determine this question.

In the same category with the want of respect for the medical body, and doubtless dependent on the same state of feelings, is the curious propensity on the part of the clergy, always manifested, to patronize, recommend, and puff all kinds of empirical measures and men. Dr. Holmes, in his amusing "Delusions of Homœopathy," gives many grotesque illustrations of this singular proclivity of ministers to run after wild humbugs and ridiculous cure-alls, as far back as Perkin's Tractors, certified to by half the reverends of New England, who were seduced by the gift of a half cent's worth of brass and pewter; and the same spirit is continued down to this morning's papers, in which the Rev. Mr. Williamson and the Rev. Mr. Drew certify to some asthmatic elixir, under their own sign manual! It is well understood that the venders of quack medicines are ready to give an extra price for a certificate from a reverend, especially if it closes with a due quantity of devout expressions o fgratitude, equally distributable to the Author of Good and the inventor of the patent medicine!

Now why do the clergy figure so prominently in the annals of quackery, even of the grossest kind? Is it because they would willingly see the educated practitioner driven into the pursuits of trade and agriculture? Or is it, that being a class of men peculiarly ignorant of the world and its ways, they are more easily misled and gammoned? Or is it because, having been taught at college a smattering of the *ologies*, they have the vanity to presume that they can make a short cut, "a royal road," to what proves so long, so arduous, so uncertain a goal, as the educated physician acknowledges his ultimatum to be, after life-long struggles and honest devotion to the truth.

The prominent defect of men, like the clergy, who have none, or only an elementary smattering in the science of medicine is this—that they insist on making a full, glorious, growing summer, of a single, solitary swallow, which they have seen, or *think they have seen*, or, at least, heard of; they ase unable to comprehend that a *post hoc* is not necessarily a *propter hoc*, that a *sequence* may not be a *consequence* —or, to use the well-known English saying respecting that class of logicians who couple things together as *cause and effect* simply because they happen about the same date, they always regard Tenterden steeple to be the undoubted cause of Godwin sands! If, perchance, they should see a patient recover after a "great medicine" of the Pottawatamies had performed his filthy orgies over him, they would never have the intrusion of one doubt that the miraculous cure resulted from the "means being blessed."

These shallow logicians never dream that the natural, spontaneous termination of nineteen-twentieths of all sickness is in recovery, and not death—that, whether treated or mal-treated, or left to the resources of the constitution, the patient may be expected to live; how much unnecessary suffering, delay and permanent mischief to the sufferer, from the omission of proper, or the administration of noxious means, being entirely a different question. When a person who is more or less sick, makes an escape, after any of the varieties of empiricism, with his life, these sagacious philosophers are ready to throw up their caps and cry *Eureka!* Many a parson who finds his child get well, somewhere about the epoch when some new quackery has been applied, goes cackling about the parish, as if the philosopher's stone had been discovered. His intelligent parishioners—finding their pastor adopting that motto so universal, as well to deserve to be emblazoned as part of the clerical arms, "*credo quia impossibile est*"—begin to ask the question whether he builds his faith in the doctrines and truths of religion, which they pay him for teaching to themselves and their families, on the same psychological processes, as those in which he hangs his medical belief. The natural conclusion is, that a man who is ready to hazard the lives of his household on practices and practitioners, in respect to whose value or safety he has less evidence than a prudent man would require of the itinerant tinker who presents himself to mend the brass kettle, is not a safe guide in matters either temporal or spiritual. Is this any explanation of the recent fact, that more than one of the clergymen of this city, who a few years since identified themselves with the now all but defunct vagaries of Hahneman, have had leave to retire to other "fields" *on account of health;* or is it that the same want of good common sense, which embracing this humbug would indicate, is merely an "*ex pede Herculum*" proof of enormous weakness and gullibility in other points of their intellectual constitution?

This train of thought has been suggested by reading an article in the last Christian Examiner, presumptively the production of some member of the clerical or pedagogic profession, on the water cure, as it is called. This guess is hazarded under no knowledge of its paternity, but for the reason that there is a solemn owlishness in its *ex cathedra* annunciation of common places about pathology—a smattering of medical ideas, such as could only be obtained from some school-girl's compend of "Physiology made Easy," or "The House I Live in"—mixed with eulogistic, self-complacent commendations, common in circulars and advertisements of "Water-cure Establishments."

Of course we have no idea of attempting a formal argument in reply to such an article, from such a source, and in such a vehicle. Nothing is more absurd than the attempt to argue down a delusion, even with an antagonist whose preliminary acquaintance with the subject matter renders it practicable to adapt reasoning to his comprehension. The endeavor to reason on a topic of medicine with a writer like this—one who manifestly is not guilty of claiming the elementary rudiments, the very A B C of acquaintance—would be as ridiculous as it would be to labor with brother Himes, or Silas Lamson, on the intricacies of exegesis, or the philological refinements of the Coptic or Sanscrit dialects with the pious end of convincing them that they are utterly ignorant of interpretation and prophecy. It would in either case be very easy to furnish the conclusive proofs, but to furnish the recipient understanding, *hic labor*, *hoc opus est!*

Some of the ridiculous points of this aquatic evangely—its analogies to various other forms of moonshine and fraud—might perhaps be made available to some innocents of both genders, who have not read enough in the earliest books of medicine to be able to comprehend the absurdities of them in a medical point of view.

Sir Gilbert Blane long ago made the remark, doubtless verified in every intelligent person's observation, that whenever a professed curer of disease, regular or empiric, undertook to set up the claim that he lost very few or no patients, it may be safely affirmed that either he was trusted with no serious cases, or would be found to lie—under a great mistake. Our cold water lacquey, who pretends to give the returns of one of these aquatic caravanseras, will find that his vaunted success is "decidedly and individually" surpassed by the "semi-occasional" announcements of Dr. Peters, Dr. Dow, and other worthies, whose cures are made "without absence from business and with no mercury," in the daily "penny medical journals." These "bastard and unlineal sons" of "Slapslapius," in their pathetic Jeremiads to the afflicted "to beware of the advertising quack across the street," report more thousands cured without being killed, than there are hundreds under the "cautious and intelligent *practitioner*" [*!!*] whose glories he celebrates, and whose veritable history and claims to confidence are foreshadowed in an official certificate of an association of his contrymen at Philadelphia, published in this Journal some months since.

Our friend Aquarius—for so bright a constellation of medical science deserves no less a personification—has apparently got hold of some crude ideas of the ancient humoral pathology, and gravely announces the theory of the water cure to consist in washing out a goodly portion of the patient, and making him over anew. Hercules, when he turned the river through the Augean stable, was the first water-cure practitioner.

Hamlet's grave-digger had only discovered, by his professional experience, that "water is a sore destroyer of your whoreson dead bodies." These modern comicalities have carried their prototype's observation still farther, and find that its virtues in decomposing the living carcass are no less wonderful. A man is washed out and made over, our learned author avers, more in six weeks under the water-cure, than in three years of the old and natural way! "It is a remarkable, but perfectly-well-authenticated fact," he says, "that drugs which have lain dormant in the system sometimes *for years*, make their appearance, clearly perceptible to the senses, and are expelled from the body during these cures." A most remarkable fact truly! the only parallel to which in history, as far as we have read, is the remarkable congelation of sound in the trumpet of a celebrated *German* practitioner—Mein Herr Baron v. Munchausen—which being subjected to the sweating processes, resulting from setting it up in the chimney corner, gave forth most melodious music; charges, marches, retreats, and triumphal blasts, were *made over* in the best conceivable style!*

Any intelligent reader of the Christian Examiner, who would see how insignificant a part these humoral notions play in modern pathology, can consult the work of Chapman on Therapeutics, or the notes by Dr. Beck to Murray's Materia Medica for the other side, or any other respectable treatise in any regular physician's library. After so doing, he will arrive at the conclusion that any

* A friend at our elbow suggests that the notion of drugs working out through the skin after long *remora* in the system, was deduced in this vicinity from the case of the Rev. Mr. J———, once a respectable minister but who abandoned himself to drink and opium eating. He was induced, by some of the cold water sages, to try the effect of their panacea. When he was under a full head of the sweating and steaming process sure enough the pent up vapors gave forth a most unsavory odor of laudanum! With their usual cautious generalization, and the modest self reliance which always marks the inductive philosopher, the old women of both sexes who watched the phenomena, at once held up their hands in amazement at the miraculous fact—more wonderful than the liquefaction of the blood of St. Januarius—that the long locked up poison was compelled to desert its victim! Poor J———! the reform was of short duration. The Lunatic Hospital at Worcester soon received him, and the amusing explanation came out, that a small vial of the precious tincture, which he had concealed about his person to comfort him in passing through Jordan—for his dread of the element had long been perfectly *hydrophobic*—had been accidentally fractured, and its perfume thus shed abroad!

"You may break, you may ruin the vase if you will,
But the scent of the roses will hang round it still!"

brain which can accept of the crudities of hydropathy, must have fully partaken of the watery imbibitions and replacements of its owner.

Were this miserable cold-water humbug—this poor quackery of using water "as his stimulant, his tonic, his purgative, his counter-irritant, &c.," applied as the seventh son or other empirics apply their panaceas to the worn-out nervous, hypochondriacal, hysterical cases, ever in search of something new, or were it like homœopathy or Broussaism, only guilty of murder by omission, its responsibility would be comparatively trifling. The fatal effects of the use of cold water in acute visceral inflammations, and indeed in serious diseases generally, are not passing without note and record by the profession; but as in Thomsonism, the aggregate of terrible results may sometime be presented to the utter confusion of pretenders, if they have shame or conscience left. The profession have found, long since, that these delusions die quickest, if let alone—which has prevented the publication of certain cases of hydropathic homicide, cruel enough to make the ears of those who hear tingle.

Were only the enervated, exhausted, transcendental victims of excesses and indolence, prostrated by pernicious personal practices, the only subjects sent to these cold water seminaries, no great harm might result. Whether the treatment of these cases of *goneness* (as they are denominated by a celebrated panaceist) is "Dr. Banning's Lace," the cold water cure, or "Brandreth's Constitution Pills," is "much of a muchness." There must be a certain portion of the community, who have a natural tendency to quackery; it always has been so and will, no doubt always continue to be so. It fortunately happens that this class is ever much smaller than it seems to be. Watch the sisters, male and female, who now spend their time in running after this hydropathic mummery. Last year they were equally full of transcendentalism, the year before of homœopathy, the years before of animal magnetism, Grahamism, phrenology. Next year they will be Fourierites communists, George Sandists, &c. The farce will be the same, only the performers will, like Othello, be a little more "declined into the vale of years"—*anglice*, old maidendom. Like the supernumeraries of a strolling theatre, who first appear as a procession of holy monks, next march as a band of Roman soldiers, then as a chorus of Alpine peasants, until the youthful spectator is lost in amazement at the legion who are hired for his entertainment, when an unlucky pair of bowlegs, or a portentous Bardolphian nasal promontory, lets the cat out of the bag. He finds, and is a little provoked to find that all the varieties he has seen are only the same performers, doubling their parts, and coming round again after a due circulation, like the moving figures, which walk out on one side of the face of a Dutch clock, when it chimes, and in at the other.

One word before we part, to our friends of the Christian Examiner—a most respectable periodical of its kind, well supported by medical as well as by other educated gentlemen. They must be intelligent enough to realize that the breach between the educated professions of medicine and theology is wide enough already, without any effort on the part of either to make the estrangement more complete. Suppose, to illustrate the feelings with which a regular physician may be supposed to cast his eye over the pages now under remark—that an educated clergymen should take up a number of this Medical Journal, and find a labored attempt to show by such plausible pretences and specious assertions, as may easily be applied to any topic whether true or absurd, that the researches of all divines, philologists, and scholars, heretofore, have been superseded by the mesmeric revelations of "Andrew Jackson Davis, the Poughkeepsie seer;" that the life-long labors of Channing, Ware, Norton, and all the galaxy of good and great men they have habituated themselves to reverence, as the guides of their lives, have been eclipsed and rendered nugatory, by the discoveries and new interpretations, "right out of their own heads," of Ascension Miller, Brother Lamson, or Elder Knapp; that the theology of Cambridge, Andover, and Princeton is all rank nonsense since the revelations in Joe Smith's "golden plates of Mormon." He would not more quickly pitch these pages behind his backlog with loathing and disgust, than would the physician, when he sees the sages of his profession, dead and living, *overslawed* and rejected in favor of the poor vagaries of an ignorant German peasant, and the humbugs of Mr. Gully (whose name is of no inapt significancy), or any other of the more educated but less sagacious, apostles of this aquatic gospel.

We would venture to remind your theological confreres, Mr. Editor, of the old and homely adage, *ne sutor*, &c.

They may take it for granted, that it is neither their "mission" or vocation—still less those of their hydrostatic correspondent, *tantas componere lites*, as must always, it is hoped, exist between the science of medicine, and the humbuggery of panaceas—between the regular physician and the empirical pretender. They should also not forget, that there are various little theological differences still pending commencing with the relations of Adam's original transgression, and continued uninterruptedly down to Dr. Bushnell's sermon before the divinity class of the University, last Sunday night. Better elaborate and discuss these topics, on which they have some education and knowledge before proceeding to those in regard to which it is no discredit to any clerical gentleman or schoolmaster to say that he knows nothing.

When all the controversies of sects are settled, then they will be excusable if they venture on the quixotic errand of aiding and relieving the distressed sisters, male and female, who are ever so anxious that people should be born and die in some approved method which can be learned without toil. Our respectful counsel, then, to our brethren of the Christian Examiner would be, to stick close to their christian labors and investigations, leaving medicine to its legitimate organs, and cold water to the engines, which will, no doubt continue a short time longer to pump forth

"Their weak, everlasting, washy flood."

Cold water, hot water, ice water, yea steam of water, are all very good, useful and comfortable appliances, directed by wise and experienced heads, and assisted by proper therapeutic and dietetic means; but that cold water can be a panacea—a cure-all for all diseases, like Brandreth's pills and Gordak's drops——can only be expected when Yankee ingenuity shall have invented that augur which shall bore a square, a round, or triangular hole, at will.

In taking our leave of the writer of this "first-rate notice" of one of the many cold-water hotels with which our vicinity is becoming *inundated*—soon, we doubt not, to be left high and dry, by that tidal reflux incident to shallow flats, and to expose a mass of corruption to the noon-day sun of common sense and judgment—we would commend to his consideration the texts of St. Paul and Solomon (his reading may perhaps recall to him the author and the phraseology), about "being wise in one's own conceit" and attending to one's own business. We hope we have done the honorable profession of the clergy no wrong in presuming the writer to belong to it, or to be a "schoolmaster abroad"—very far off from home indeed. We opine the latter to be his vocation, from the peculiarly self-satisfied, dogmatic air which men accustomed to throw out their opinions before children, without hazard of reputation, naturally assume. If, on the other hand, he is a clergyman, it is most probably of some of the new fangled transcendental varieties. His hortations about the religious and moral duty of swallowing down this kind of wet-sheet baptism, are announced in that tone of holy snuffling, with which the devout Musselman peddles his wares, in the streets of Constantinople; "In the name of the prophet—*figs!*" His mixture of quackery and cant reminds us strongly of the directions in the envelope of a bottle of "Dr. ———'s acoustic oil for the certain cure of deafness" viz., to use the oil daily, to abstain from labor on the Sabbath, and bath the privates in cold water! In some of our amphibious prophet's school books, if that be the respectable vocation from which he has stepped aside for less respectable labor, he will find some verses, the hydraulic imagery of which we may commend to his fructification, ending thus—

"*Drink deep,* or taste not, the Pierian spring,
For *shallow draughts* intoxicate the brain,
But *drinking largely* sobers us again."

